# Sirtuin 3 Deficiency Aggravates Kidney Disease in Response to High-Fat Diet through Lipotoxicity-Induced Mitochondrial Damage

**DOI:** 10.3390/ijms23158345

**Published:** 2022-07-28

**Authors:** Monica Locatelli, Daniela Macconi, Daniela Corna, Domenico Cerullo, Daniela Rottoli, Giuseppe Remuzzi, Ariela Benigni, Carlamaria Zoja

**Affiliations:** Istituto di Ricerche Farmacologiche Mario Negri IRCCS, Centro Anna Maria Astori, Science and Technology Park Kilometro Rosso, 24126 Bergamo, Italy; daniela.macconi@marionegri.it (D.M.); daniela.corna@marionegri.it (D.C.); domenico.cerullo@marionegri.it (D.C.); daniela.rottoli@marionegri.it (D.R.); giuseppe.remuzzi@marionegri.it (G.R.); ariela.benigni@marionegri.it (A.B.); carlamaria.zoja@marionegri.it (C.Z.)

**Keywords:** high-fat-diet, sirtuin 3, oxidative stress, mitochondrial damage, lipotoxicity, kidney damage

## Abstract

Sirtuin 3 (SIRT3) is the primary mitochondrial deacetylase that controls the antioxidant pathway and energy metabolism. We previously found that renal *Sirt3* expression and activity were reduced in mice with type 2 diabetic nephropathy associated with oxidative stress and mitochondrial abnormalities and that a specific SIRT3 activator improved renal damage. SIRT3 is modulated by diet, and to assess whether *Sirt3* deficiency aggravates mitochondrial damage and accelerates kidney disease in response to nutrient overloads, wild-type (WT) and *Sirt3*^−/−^ mice were fed a high-fat-diet (HFD) or standard diet for 8 months. *Sirt3*^−/−^ mice on HFD exhibited earlier and more severe albuminuria compared to WT mice, accompanied by podocyte dysfunction and glomerular capillary rarefaction. Mesangial matrix expansion, tubular vacuolization and inflammation, associated with enhanced lipid accumulation, were more evident in *Sirt3*^−/−^ mice. After HFD, kidneys from *Sirt3*^−/−^ mice showed more oxidative stress than WT mice, mitochondria ultrastructural damage in tubular cells, and a reduction in mitochondrial mass and energy production. Our data demonstrate that *Sirt3* deficiency renders mice more prone to developing oxidative stress and mitochondrial abnormalities in response to HFD, resulting in more severe kidney diseases, and this suggests that mitochondria protection may be a method to prevent HFD-induced renal injury.

## 1. Introduction

Metabolic syndrome, which is a cluster of concurrent clinical conditions, including obesity, dyslipidemia, hyperglycemia and hypertension, is emerging as a global health threat in part due to the spread of Western diets worldwide and the increased prevalence of obesity in adults and the young population [1,2]. Individuals with metabolic syndrome are at high risks of developing type 2 diabetes, cardiovascular diseases and chronic kidney disease [2,3,4].

There is experimental evidence that high-fat-diet (HFD) feeding in rodents induces systemic metabolic abnormalities, which mimic those observed in patients with metabolic syndrome [5,6]. After the onset of metabolic syndrome, mice on an HFD also exhibited renal injury characterised by albuminuria and renal histological changes, such as glomerular extracellular matrix accumulation and basement membrane thickening, which were accompanied by oxidative stress and inflammation [7].

Metabolic diseases are associated with the dysfunction of the mitochondria, an important site of intermediary metabolism within the cell [8]. Post-translational modifications of mitochondrial proteins, and in particular lysine acetylation, are a key feature in regulating mitochondrial function to maintain cellular homeostasis [9]. Overall acetylation levels are controlled in part by the sirtuin family of nicotinamide adenine dinucleotide (NAD)^+^-dependent protein deacetylases, where sirtuin 3 (SIRT3) is the primary mitochondrial deacetylase [10,11,12]. Numerous reports have shown that SIRT3 regulates mitochondrial function and maintains redox homeostasis by targeting mitochondrial enzymes involved in metabolic pathways, including the tricarboxylic acid (TCA) cycle, the urea cycle and fatty acid β-oxidation, and in the antioxidant response system [13,14]. Calorie intake regulates mitochondrial function through the modulation of SIRT3, which acts as an important metabolic sensor tasked with restoring cell homeostasis under stress. The expression of SIRT3 is downregulated by nutrient excess and, conversely, is increased in response to caloric restriction [15,16]. Indeed, *Sirt3* mRNA and protein expression are reduced in skeletal muscles and in the livers of mice made obese by a chronic HFD, thus causing mitochondrial protein hyperacetylation, which contributes to the development of insulin resistance and metabolic syndrome [17,18,19]. We previously showed that, in BTBR *ob*/*ob* mice that develop type 2 diabetic nephropathy, renal *Sirt3* mRNA expression and activity were reduced and associated with increased oxidative stress and mitochondrial structure and function abnormalities. The selective activation of SIRT3 through the administration of honokiol, a natural biphenolic compound, ameliorated renal damage by preserving mitochondrial wellness [20].

In the present study, we investigated whether *Sirt3* deficiency in mice could aggravate mitochondrial damage and accelerate kidney disease in response to chronic HFD feeding.

## 2. Results

### 2.1. Laboratory and Systemic Parameters

**Figure 1:**
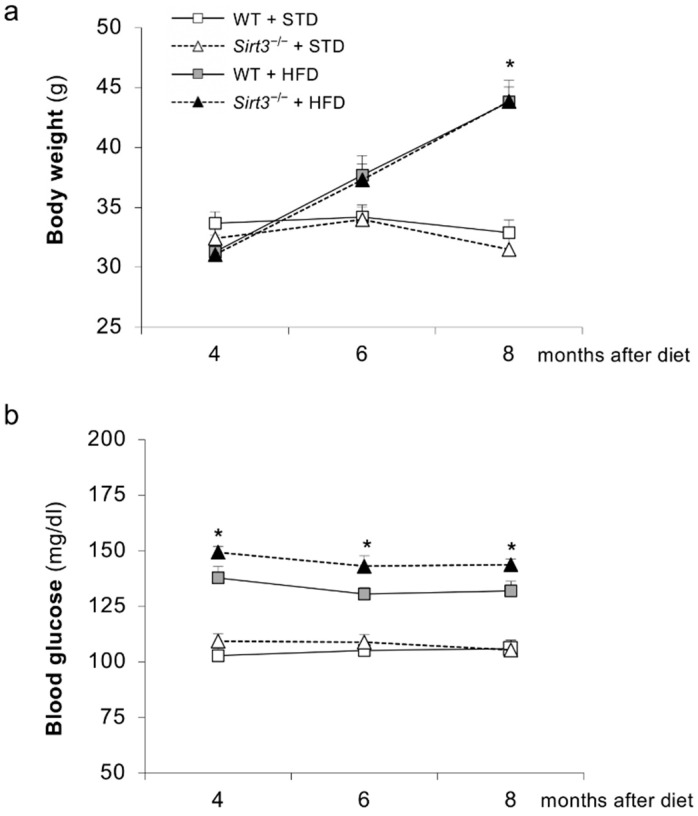
Systemic and laboratory parameters in WT and *Sirt3*^−/−^ mice in each dietary group. Time course of (**a**) body weight and (**b**) blood glucose levels in WT and *Sirt3*^−/−^ mice on standard (STD) or high-fat-diet (HFD). Data are mean ± SEM and were analyzed by one-way ANOVA followed by Tukey’s multiple comparisons test, * *p* < 0.001 versus corresponding group fed the standard diet.

Figure 1 and Table 1 report laboratory and systemic parameters measured in WT and *Sirt3*^−/−^ mice fed an HFD or a standard diet during the 8-month study period.

Both groups of mice fed HFD exhibited an increase in body weight compared with the corresponding groups given a standard diet, which reached statistical significance 8 months after diet feeding. No difference in body weight was observed between WT and *Sirt3*^−/−^ mice on HFD (Figure 1a). The HFD groups developed hyperglycemia, unlike the standard diet groups, throughout the entire experimental period (Figure 1b). The HFD-induced hyperglycemia in WT and *Sirt3*^−/−^ mice was comparable.

Plasma cholesterol and triglyceride levels were significantly higher in the WT and *Sirt3*^−/−^ mice on HFD compared to the corresponding groups fed the standard diet (Table 1). Systolic blood pressure levels were similar in all experimental groups. An increase in heart rate was recorded in *Sirt3*^−/−^ mice fed with HFD, compared to both WT mice fed with HFD and to *Sirt3*^−/−^ mice on the standard diet. Notably, in a previous study, we showed that *Sirt3*^−/−^ mice—which were fed the standard diet—experienced cardiac damage with aging, i.e., after 20 months of age [21]. Here, the HFD anticipated the development of signs of cardiac damage in 10-month-old *Sirt3*^−/−^ mice. Renal function, measured as BUN, was comparable across experimental groups (Table 1).

### 2.2. Sirt3 Deficiency Accelerates and Exacerbates HFD-Induced Albuminuria

**Figure 2:**
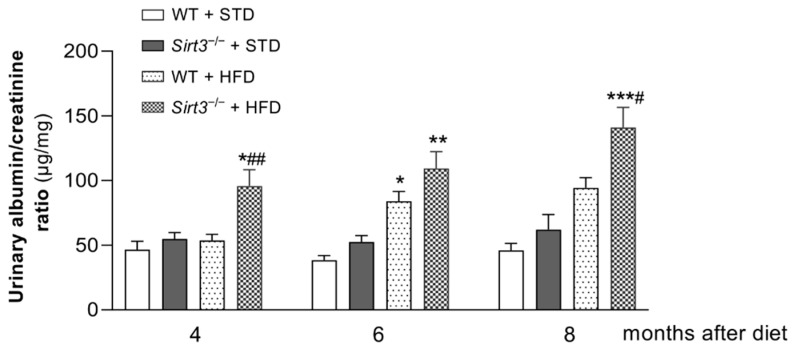
*Sirt3* deficiency exacerbates urinary albumin excretion in mice on HFD. Time course of urinary albumin to creatinine ratio in WT and *Sirt3*^−/−^ mice fed with a standard diet or HFD. Data are mean ± SEM and were analyzed by one-way ANOVA followed by Tukey’s multiple comparisons test, * *p* < 0.01, ** *p* < 0.001, *** *p* < 0.0001, versus corresponding group treated with standard diet; ^#^ *p* < 0.05, ^##^ *p* < 0.01 versus WT + HFD.

The time course of albuminuria, measured as urinary albumin-to-creatinine ratio (UACR), in WT and *Sirt3*^−/−^ mice fed with HFD or standard diets is shown in Figure 2. In WT mice, the HFD caused a significant increase in UACR levels compared to the standard diet, starting from 6 months after HFD feeding. Unlike WT mice, *Sirt3*^−/−^ mice had already developed albuminuria at 4 months after HFD, which increased further over time. Notably, in response to HFD, urinary albumin excretion was significantly higher in *Sirt3*^−/−^ than in WT mice, indicating that the lack of *Sirt3* made mice more susceptible to developing early and more severe renal disease.

### 2.3. Sirt3 Deficiency Worsens Glomerular and Tubular Damage and Increases Renal Inflammation in Mice Fed HFD

**Figure 3:**
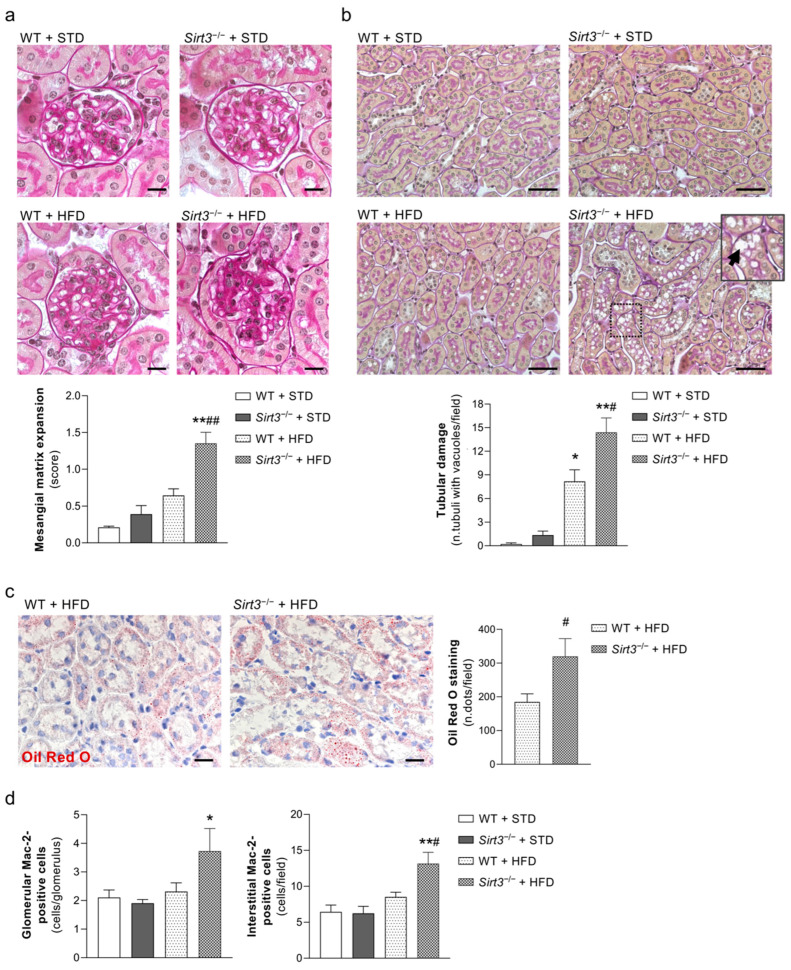
*Sirt3* deficiency worsens glomerular and tubular damage, lipid accumulation and renal inflammation in mice on a HFD. (**a**) Periodic acid–Schiff–stained (PAS) sections of representative glomeruli, and mesangial matrix quantification in WT and *Sirt3*^−/−^ mice fed with a standard diet or HFD for 8 months. Scale bars: 20 μm. (**b**) PAS sections of representative tubular fields and quantification of tubular damage from WT and *Sirt3*^−/−^ mice fed STD or HFD for 8 months. Scale bars: 50 μm. HFD caused vacuolization in most proximal tubule cells (inset, arrow). (**c**) Representative tubular fields and quantification of Oil Red O staining from WT and *Sirt3*^−/−^ mice fed with HFD. Scale bars: 20 μm. (**d**) Quantification of glomerular and interstitial accumulation of Mac-2-positive monocytes/macrophages in WT and *Sirt3*^−/−^ mice fed STD or HFD. Data are mean ± SEM and were analyzed by one-way ANOVA followed by Tukey’s multiple comparisons test, or Student’s *t* test, * *p* < 0.05, ** *p* < 0.001 vs corresponding groups with standard diet, ^#^ *p* < 0.05, ^##^ *p* < 0.01 vs. WT + HFD.

The histological analysis of PAS-stained kidney sections revealed a mild glomerular lesion consisting of mesangial matrix expansions in WT mice fed with HFD but it was not found in WT mice fed with a standard diet. The extent of glomerular damage increased substantially in *Sirt3*^−/−^ mice fed with HFD, as demonstrated by a significant increase in mesangial matrix expansion compared with both WT mice on HFD and *Sirt3*-deficient mice fed with a standard diet (Figure 3a). In response to HFD, *Sirt3*-deficient mice exhibited more extensive vacuolisation in proximal tubular cells compared to WT mice (Figure 3b). Lipid accumulations in the proximal tubules, as evaluated with Oil Red O staining, were also more prominent in *Sirt3*^−/−^ mice (Figure 3c).

As shown in Figure 3d, *Sirt3*^−/−^ mice on HFD exhibited a marked accumulation of Mac-2-positive monocytes/macrophages in either the glomeruli or the renal interstitium, unlike WT mice fed with HFD, which did not show renal inflammation.

### 2.4. Sirt3 Deficiency Alters the Glomerular Filtration Barrier in Mice Fed HFD

**Figure 4:**
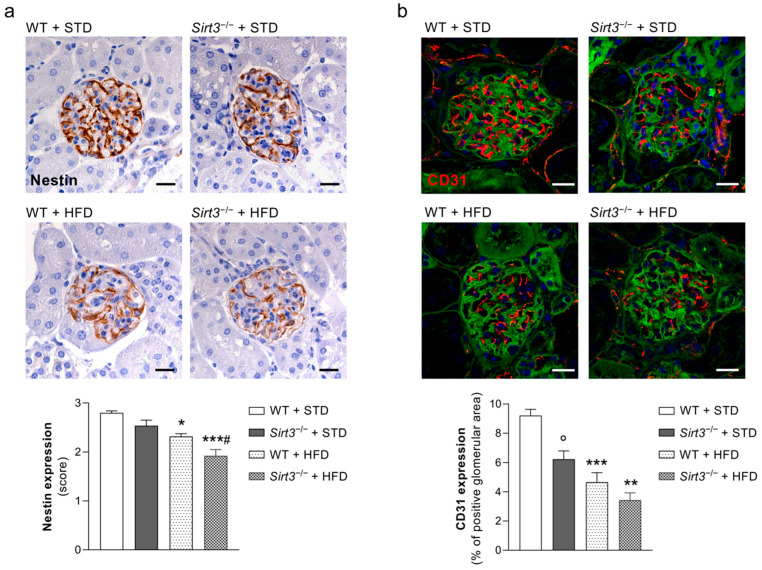
*Sirt3* deficiency causes podocyte dysfunction and reduced glomerular endothelial cell density in mice on HFD. (**a**,**b**) Representative images and quantification of glomerular nestin expression (**a**), markers of podocyte dysfunction, and of CD31 (**b**), an endothelial cell marker, in WT and *Sirt3*^−/−^ mice fed standard diet or HFD. Data are mean ± SEM and were analyzed by one-way ANOVA followed by Tukey’s multiple comparisons test, * *p* < 0.05, ** *p* < 0.01, *** *p* < 0.001 versus corresponding group treated with standard diet; ^#^ *p* < 0.05 vs. WT + HFD; ° *p* < 0.01 vs. WT + STD.

Since albuminuria develops as a consequence of impaired glomerular filtration barrier permselective function, we explored whether the worsening effect of *Sirt3* deficiency on urinary albumin excretion in response to HFD could be related to functional and/or structural changes in podocytes and glomerular endothelial cells, which are the main determinants of the glomerular filtration barrier [22,23]. Our analysis of nestin, a protein found in the podocyte cytoskeleton, which plays an important role in maintaining podocyte function [24], revealed reduced staining in the glomeruli of WT mice fed with HFD instead of a standard diet. The decrease in nestin expression in response to HFD was even more evident in mice deficient for *Sirt3* to the extent that a significant difference between *Sirt3*^−/−^ and WT mice was observed (Figure 4a).

To investigate the effect of *Sirt3* deficiency on glomerular endothelial cells, we performed immunohistochemical analysis for CD31, a specific marker of endothelial cells. WT mice on HFD exhibited a significant reduction in glomerular CD31 staining compared to WT mice fed with the standard diet (Figure 4b). The effect of HFD in causing glomerular capillary rarefaction was worsened further by *Sirt3* deficiency, and there was a statistical difference between the two groups. In response to HFD, *Sirt3*-deficient mice exhibited lower glomerular CD31 expression than the corresponding animals fed with a standard diet. The extent of the reduction was similar to that observed in WT mice. However, a significant reduction in CD31 staining was already found in glomeruli from *Sirt3^−/−^* mice fed with a standard diet compared to WT mice, indicating that a lack of *Sirt3* favours glomerular capillary rarefaction.

### 2.5. Sirt3 Deficiency Aggravates HFD-Induced Oxidative Stress and Mitochondrial Damage

**Figure 5:**
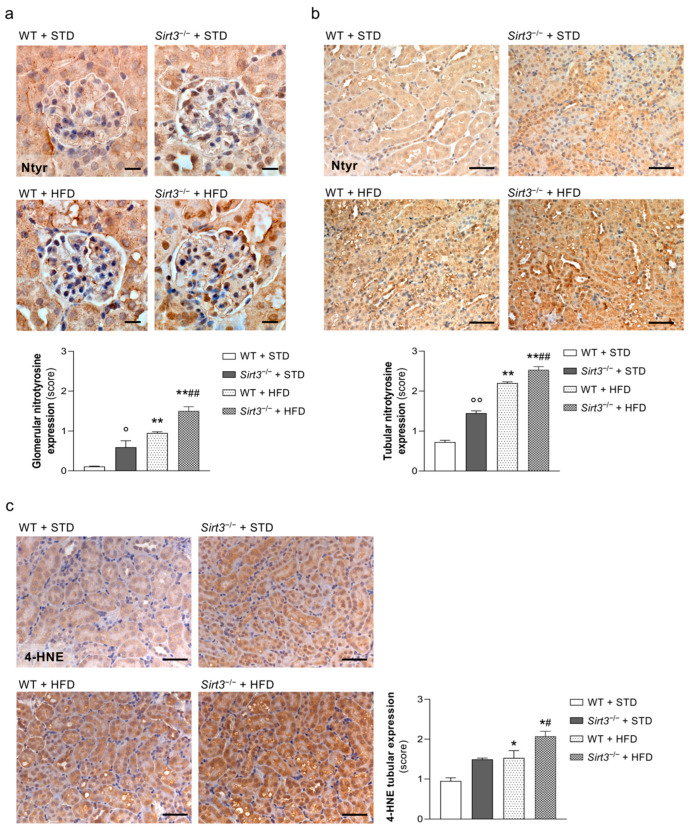
*Sirt3* deficiency favours HFD-induced oxidative stress. (**a**,**b**) Representative images and quantification of glomerular (**a**) and tubular (**b**) nitrotyrosine expression in WT and *Sirt3*^−/−^ mice fed standard diet or HFD for 8 months. Scale bars: 20 μm (**a**) and 50 μm (**b**). (**c**) Representative images and quantification of tubular 4-HNE expression in WT and *Sirt3*^−/−^ mice fed with a standard diet or HFD. Scale bars: 50 μm. Data are mean ± SEM and were analyzed by one-way ANOVA followed by Tukey’s multiple comparisons test, * *p* < 0.05, ** *p* < 0.0001 versus corresponding group treated with standard diet; ^#^ *p* < 0.05, ^##^ *p* < 0.01 vs. WT + HFD; ° *p* < 0.05, °° *p* < 0.0001 vs. WT + STD.

A high-fat-diet has been recently reported to induce renal oxidative stress and mitochondrial dysfunction [25]. As SIRT3 plays an important role in regulating cellular antioxidant pathways, we investigated whether a *Sirt3* deficiency makes mice on HFD more prone to exhibiting oxidative stress and mitochondrial abnormalities. The expression of nitrotyrosine, a marker of peroxynitrite production and the reaction product of nitric oxide and superoxide anion, was significantly higher in the glomeruli and in the tubules of WT mice fed with HFD compared with mice on a standard diet. Notably, glomerular and tubular nitrotyrosine staining were significantly higher in *Sirt3*-deficient mice than in WT mice fed with either a standard diet or HFD (Figure 5a,b). Moreover, the expression of 4-hydroxynonenal (4-HNE), an end-product of lipid peroxidation and a marker of oxidative stress, increased in the renal tubules of WT mice fed with HFD compared with mice on a standard diet, and this alteration was even more marked in *Sirt3*^−/−^ mice that exhibited a significantly higher expression of 4-HNE than WT mice (Figure 5c).

**Figure 6:**
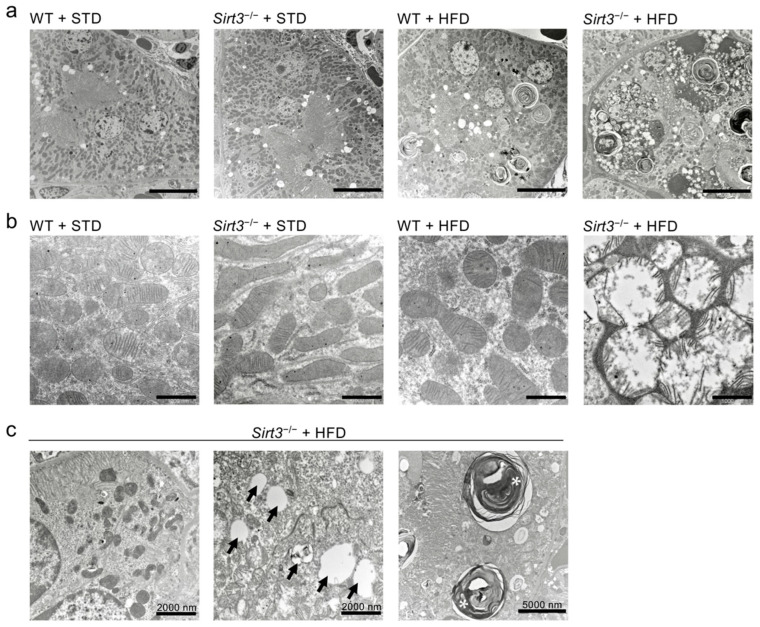
*Sirt3* deficiency worsens HFD-induced mitochondrial damage. (**a**) Representative micrographs show electron microscope images of proximal tubular cells from WT and *Sirt3*^−/−^ mice fed with a standard diet or HFD. Scale bars: 10,000 nm. (**b**) Representative electron microscope images show mitochondrial swelling with disarrangement of cristae only in *Sirt3*^−/−^ mice fed with HFD. Scale bars: 1000 nm. (**c**) *Sirt3*^−/−^ mice fed with HFD show different ultrastructural alterations as an altered mitochondrial distribution, lipid droplets (arrows) and myelin figures (asterisks).

Concomitant with the increased oxidative stress observed in tubular cells, HFD caused clear mitochondrial damage in *Sirt3*^−/−^ mice, which is characterised by matrix swelling and a disarrangement of the cristae (Figure 6a,b). In some proximal tubular cells, mitochondria appeared to be scattered in the cytoplasm without alignments and orientations (Figure 6c). In WT mice fed with HFD, mitochondrial abnormalities were less frequent. In response to HFD, ultrastructural analysis also revealed the presence of numerous lipid droplets and myelin figures in the cytoplasm of proximal tubular epithelial cells from both WT and *Sirt3*^−/−^ mice (Figure 6a,c). These specific alterations were not present in the groups of mice fed with a standard diet.

**Figure 7:**
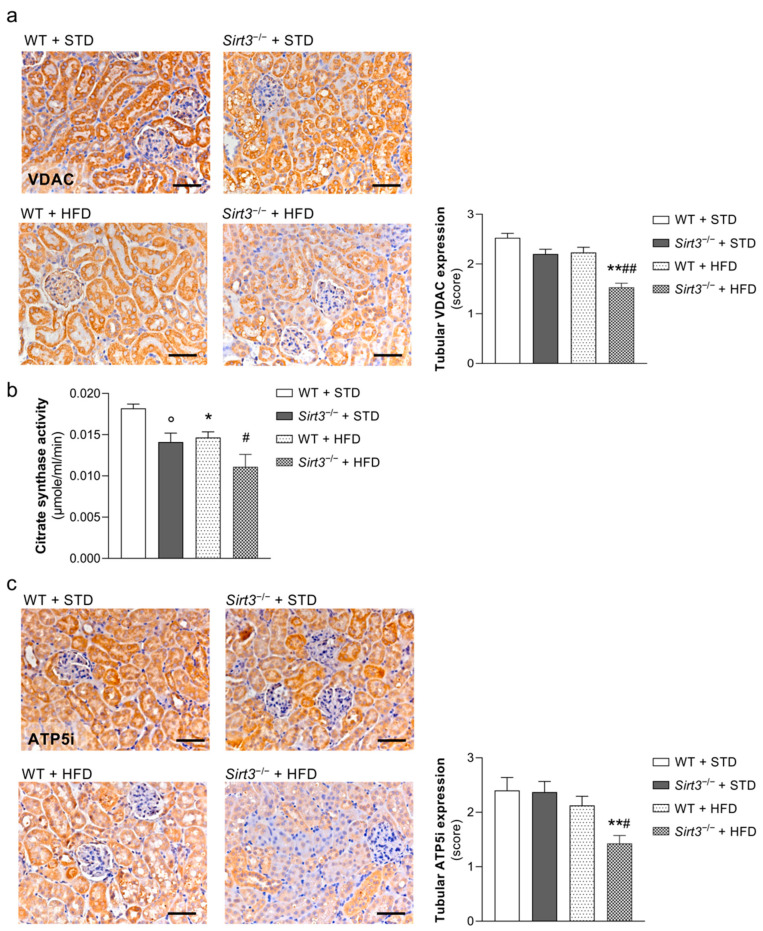
*Sirt3* deficiency affects mitochondrial mass and integrity in mice on a HFD. (**a**) Representative images and quantification of VDAC expression in renal tubules from WT and *Sirt3*^−/−^ mice fed with a standard diet or HFD for 8 months. (**b**) Levels of citrate synthase activity in renal tissue of WT and *Sirt3*^−/−^ mice fed with a standard diet or HFD. (**c**) Representative images and quantification of ATP5i expression in renal tubules from WT and *Sirt3*^−/−^ mice fed with a standard diet or HFD for 8 months. Scale bars: 50 μm. Data are mean ± SEM and were analyzed by one-way ANOVA followed by Tukey’s multiple comparisons test, * *p* < 0.05, ** *p* < 0.01 vs corresponding group treated with standard diet; ^#^ *p* < 0.05, ^##^ *p* < 0.001 vs. WT + HFD; ° *p* < 0.05 vs. WT + STD (unpaired Student’s *t*-test).

Since proximal tubules rely on the mitochondrial oxidative metabolism for the energy supply needed to function [26], we investigated whether *Sirt3* deficiency in mice fed with HFD resulted in alterations in mitochondrial proteins that regulate the function and structure of these organelles. Specifically, we analysed the expression of the mitochondrial marker voltage-dependent anion channel (VDAC), the most abundant protein of the outer membrane [27]. While no difference in VDAC expression was observed in WT mice fed with HFD compared to a standard diet, *Sirt3^−/−^* mice on HFD exhibited a significant reduction in VDAC expression compared to WT mice fed with HFD (Figure 7a), indicating a decrease in mitochondrial mass. Next, we measured the activity of citrate synthase, an enzyme that catalyzes the first step of TCA cycle [28] and a marker of mitochondrial mass and integrity [21]. The levels of citrate synthase activity were reduced in WT mice fed with HFD compared with WT mice fed with a standard diet. *Sirt3^−/−^* deficiency resulted in lower levels of enzymatic activity than WT mice fed with a standard diet, which were further reduced after HFD (Figure 7b). Mitochondrial alterations paralleled changes in the mitochondrial energy metabolism, as revealed by the downregulation of ATP5i, a subunit of the H+-ATP synthase and the very last enzyme in the oxidative phosphorylation pathway responsible for the synthesis of ATP driven by a proton gradient across the inner mitochondrial membrane [29]. As shown in Figure 7c, ATP5i was markedly reduced in the proximal tubular cells of *Sirt3^−/−^* mice after being fed with HFD, unlike WT mice, which exhibited no changes in protein expression.

## 3. Discussion

The present study demonstrates that *Sirt3* deficiency is a predisposing factor for the aggravation of oxidative stress and mitochondrial abnormalities, leading to worsening kidney damage in response to chronic HFD feeding.

*Sirt3*-deficient mice fed with HFD developed earlier and more severe albuminuria than HFD-fed WT mice, which could reflect more severe alterations of the glomerular filtration barrier, as demonstrated by the lower expression of the podocyte structure protein nestin and extensive glomerular capillary rarefaction. These results are in line with our previous findings with regard to the kidneys of BTBR *ob*/*ob* diabetic mice, where impaired glomerular SIRT3 deacetylase activity was associated with albuminuria and podocyte and endothelial dysfunction and loss [20]. More severe histological lesions characterised by the glomerular accumulation of extracellular matrix and tubular vacuolisation developed in the kidneys of *Sirt3*^−/−^ mice in response to the HFD, concomitant with enhanced lipid accumulations.

There is growing evidence that HFD causes the dysregulation of renal lipid metabolisms, which promote the accumulation of lipids in the kidney, leading to renal injury [30,31]. Lipotoxicity driven by ectopic lipid accumulation in the kidney has been recognised as having a causal role in renal dysfunction in several pathological conditions, including metabolic syndrome, obesity-related glomerulopathy and diabetic nephropathy [32,33,34]. The kidney is a highly metabolically active organ that relies on fatty acid mitochondrial β-oxidation for its function as the preferred metabolic pathway for ATP production [35]. SIRT3 increases mitochondrial fuel supplies through the deacetylation of long-chain acylcoenzyme A dehydrogenase (LCAD), which regulates fatty acid β-oxidation [36], and there is evidence that LCAD was hyperacetylated in the liver of *Sirt3*-deficient mice fed with HFD that had severe hepatic steatosis, consistent with the reduced fatty-acid oxidation [19]. Excess fatty acids, which do not undergo mitochondrial oxidation, can be esterified into triglycerides and stored as lipid droplets. Our study confirms the presence of lipid droplets mostly in renal tubules in response to HFD, which increased markedly in the absence of *Sirt3.* In keeping with this, extensive fatty acid deposition related to impaired fatty-acid oxidation has been described in the kidneys of mice with cisplatin-induced acute kidney injury, which was modulated by SIRT3. Specifically, the activation of SIRT3 through honokiol treatment regulated fatty-acid oxidation via the deacetylation of liver kinase B1 and the activation of AMP-activated protein kinase [37]. Lipid excess in the kidney contributes to the progression of renal disease through several mechanisms including the activation of an inflammatory response [38]. Consistently, the increased accumulation of monocytes/macrophages was found in the kidneys of *Sirt3*^−/−^ mice given the HFD, mostly in the renal interstitium, which could have contributed to kidney damage. Our findings are in line with the evidence that free fatty acids can induce ROS-related inflammation in renal tubules, which is attenuated by SIRT3 [39].

SIRT3 is an important regulator of redox homeostasis and a deficiency for SIRT3 results in impaired antioxidant defences, which cannot counteract the harmful effects of ROS generation [13,14]. Here, an important finding is that *Sirt3*-deficient mice fed an HFD developed more oxidative stresses in the kidney than WT mice. The observation of increased peroxynitrite generation in the glomeruli of these mice is in line with our previous report on experimental type 2 diabetic nephropathies, where reduced renal *Sirt3* expression was associated with the hyperacetylation of the antioxidant enzyme superoxide dismutase 2 and a concomitant increase in glomerular oxidative stress and mitochondrial abnormalities mainly found in podocytes [20]. These defects were corrected by honokiol, a specific SIRT3 activator, which also limited podocyte dysfunction [20].

The enhanced oxidative stress observed in response to HFD and further reinforced by *Sirt3* deficiency was not confined to glomeruli but also observed in renal tubules, as demonstrated by the increased generation of peroxynitrite and the accumulation of lipid peroxidation product 4-HNE. Because of its electrophilic nature, 4-HNE can form protein adducts with cysteine, histidine, arginine and lysine amino acid residues of proteins, leading to their inactivation [40]. Notably, several mitochondrial proteins involved in protein import, oxidative phosphorylation and TCA cycle, some of which are regulated by SIRT3, are targets of 4-HNE modification [41,42]. SIRT3 itself, which is localised in the inner mitochondrial membrane, can be exposed to high concentrations of 4-HNE, which inhibits SIRT3 activity via thiol-specific modifications [43]. Another important finding of this study is that excessive oxidative stress in renal tubules of *Sirt3*-deficient mice fed the HFD paralleled ultrastructural abnormalities of mitochondria consisting of swollen organelles and irregular cristae. In these animals, we also observed reductions in tubular VDAC, citrate synthase activity and ATP5i, which may contribute to the very severe loss in the integrity of the mitochondrial structure and energy depletion in response to HFD. Indeed, VDAC is a multi-functional channel that plays a crucial role in coordinating the metabolic and energetic functions of mitochondria and in regulating mitochondrial permeability and membrane potential [27]. Citrate synthase is involved in mitochondrial oxidative metabolism, and it is known that its activity is impaired in *Sirt3*-deficient mice, resulting in reduced mitochondrial density [21]. On the other hand, ATP5i is a subunit of mitochondrial ATP synthase, which regulates the assembly of ATP synthase dimers, leading to the formation of mitochondrial cristae, which are essential for the synthesis of ATP through oxidative phosphorylation pathways [44]. These findings indicate the important role SIRT3 plays in maintaining mitochondrial integrity and energy metabolism when exposed to excess nutrients. In line with this, a previous study has demonstrated that treatments with a tetrapeptide that targets cardiolipin and protects mitochondrial cristae structure were able to preserve mitochondrial architecture in the kidney cells of mice during HFD, thereby preventing glomerular and tubular damage [45].

In conclusion, here, we have shown that *Sirt3*-deficient mice are susceptible to experiencing severe mitochondrial abnormalities in response to HFD, which impact negatively albuminuria and glomerular and tubular damage. Our data highlight the role that SIRT3 plays as a potential therapeutic target for preserving mitochondrial integrity and the energy metabolism in response to nutrient overload and strongly support the hypothesis that mitochondria protection may be a method for preventing HFD-induced renal injury.

## 4. Materials and Methods

### 4.1. Experimental Design

Male C57BL/6J mice (wild-type or *Sirt3*^−/−^) were obtained from Envigo (Bresso, Milan, Italy) and kept in a specific pathogen-free facility at a constant temperature on a 12:12 h light–dark cycle with free access to diet and water. At 5–7 weeks of age, wild-type (WT) or *Sirt3*^−/−^ mice in the diet protocol were divided into 4 groups: WT + standard diet (*n* = 10), *Sirt3*^−/−^ + standard diet (*n* = 12), WT + HFD (*n* = 10) and *Sirt3*^−/−^ + HFD (*n* = 14). Animals were studied throughout the 8 months of feeding. The fat components in the diet were chosen according to the available data in the literature. The HFD composition was 60% of total kcal from fat, consisting of 23.5% protein, 27.3% carbohydrates and 34.3% fat (MD.06414, Envigo). The chow used as the standard control diet consisted of 18.6% protein, 44.2% carbohydrates and 6.2% fat (2018S, Envigo).

At 2-month intervals, mice were housed in metabolic cages for 24 h urine collection for albuminuria assessments. Blood samples were collected for glucose, serum blood urea nitrogen, cholesterol and triglyceride level measurements. Mice were euthanised through CO_2_ inhalation and their kidneys were collected and processed for analysis.

### 4.2. Blood and Urine Analysis

Blood glucose levels were assessed with a reflectance meter (One-Touch UltraEasy, LifeScan, Milan, Italy). Plasma cholesterol, triglycerides and serum blood urea nitrogen were measured using the Reflotron test (Roche Diagnostic Corporation, Indianapolis, IN, USA). Urinary albumin excretion was measured with the enzyme-linked immunosorbent assay (ELISA) test using the Bethyl test kit (Bethyl Laboratories Inc., Montgomery, TX, USA). Urinary creatinine concentration was measured using the enzymatic method with a Miura One autoanalyser (I.S.E. srl, Rome, Italy).

### 4.3. Systolic Blood Pressure

Systolic blood pressure was measured with a computerised tail-cuff system in conscious mice (BP-2000 Blood Pressure Analysis System, Visitech System; Apex, White Oak, NC, Tokyo, Japan).

### 4.4. Renal Histology

Duboscq-Brazil-fixed, 3 μm-thick sections of paraffin-embedded kidney were stained with periodic acid-Schiff’s (PAS) reagent (Bio Optica, Milan, Italy). To evaluate the extent of glomerular lesions, an average of 30 glomeruli per animal was examined. The degree of glomerular mesangial matrix expansion was quantified using a score between 0 and 3 (0 = no mesangial matrix expansion; 1 = minimal; 2 = moderate; 3 = diffuse mesangial matrix expansion). Tubular damage, evaluated as cytoplasmic vacuolation in proximal tubular cells, was examined in 10 fields per section (original magnification ×400) and was expressed as the number of tubules with vacuoles per field. All biopsies were reviewed by a blinded pathologist. Samples were examined using ApoTome (Zeiss, Oberkochen, Germany).

### 4.5. Oil Red O Analysis

For lipid staining, 3 μm frozen-kidney sections were fixed with 10% formalin for 5 min, rinsed with distilled water, immersed in 100% 1,2-propanediol (catalog 398039, Sigma Alderich, St. Louis, MO, USA) for 5 min and stained with Oil Red O for 9 min at 60 °C. The sections were subsequently immersed in 85% 1,2-propanediol for 5 min and washed with distilled water. Then, the sections were stained for 3 min with Mayer’s hematoxylin (Bio Optica), washed with distilled water and mounted with an aqueous solution. Twenty-five fields per animal were randomly acquired using bright-field microscopy (ApoTome). Fiji ImageJ Software (http://imagej.net/Fiji (accessed on 13 September 2021)) was used for the quantification of the number of lipid droplets per field (original magnification ×630).

### 4.6. Immunohistochemistry

For immunoperoxidase experiments, formalin-fixed, 3 μm paraffin-embedded kidney sections were incubated with Peroxidazed 1 (catalog PX968H, Biocare Medical, Pacheco, CA, USA) to quench endogenous peroxidase, after antigen retrieval in a decloaking chamber with Rodent decloaker buffer (catalog RD913M, Biocare Medical). After blocking for 30 min with Rodent Block M (catalog RBM961G, Biocare Medical), sections were incubated with rabbit anti-nitrotyrosine (catalog 06-284, Merck Millipore, Burlington, MA, USA, 1:100), rabbit anti 4-HNE (ab46545, Abcam, Cambridge, UK, 1:50), mouse anti-VDAC (ab186321, Abcam, 1:200), rabbit anti-ATP5i (HPA035010, Millipore Sigma, Burlington, MA, USA, 1:200), rat anti-Mac-2 (clone M3/38, Cedarlane, Burlington, ON, Canada, 1:600) and rat anti-mouse nestin (ab81462, Abcam, Cambridge, UK, 1:300) antibodies, followed by Rabbit on Rodent HRP-Polymer or Mouse on Mouse HRP-Polymer or Rat on Mouse HRP-Polymer (catalog RMR622G, catalog MM620 and catalog RT517, Biocare Medical) for 30 min at room temperature. Stainings were visualised using diaminobenzidine (catalog BDB2004H, Biocare Medical) substrate solutions. Slides were counterstained with Mayer’s hematoxylin (catalog MHS80-2.5L, Bio Optica, Milan, Italy), mounted with the Eukitt mounting medium (catalog 09-00250, Bio Optica) and finally observed using light microscopy (ApoTome, Axio Imager Z2, Zeiss). Negative controls were obtained by omitting the primary antibody on adjacent sections. Glomerular nitrotyrosine staining was quantified with a semiquantitative score between 0 and 3 (0: absent staining; 1: weak staining; 2: moderate staining; 3: intense staining in podocytes and endothelial cells). At least 15 glomeruli per animal were randomly analysed. Tubular nitrotyrosine, 4-HNE, VDAC and ATP5i were quantified with a semiquantitative score between 0 and 3 in tubules (0: absent staining; 1: weak staining; 2: moderate staining; 3: intense staining). At least 10–15 fields/sections for each animal were randomly analysed (original magnification, ×400). Mac-2-positive monocyte/macrophages within glomeruli were counted in a minimum of 20 glomerular cross-sections and expressed as the average number of cells per glomerulus. Mac-2-positive cells in the renal interstitium were expressed as the average number of cells per field (original magnification, ×400). Glomerular nestin staining was quantified with a semiquantitative score between 0 and 3 (0–0.5: absent or weak staining in a few podocytes; 1: altered distribution and weak staining; 2: altered distribution and moderate staining; 3: intense podocyte staining). At least 15–20 glomeruli/section for each animal were randomly analysed.

OCT-frozen kidney sections were fixed with acetone, blocked in 1% bovine serum albumin (BSA) and then incubated with the rat anti-mouse CD31 (catalog 550274, BD Pharmingen, San Jose, CA, USA, 1:100) followed by Cy3-conjugated secondary antibody (Jackson ImmunoResearch Laboratories, Cambridge, UK). Nuclei were stained with DAPI and the renal structure with FITC-wheat germ agglutinin lectin (Vector Laboratories, Burlingame, CA, USA). Negative controls were obtained by omitting the primary antibody on adjacent sections. Samples were examined using an inverted confocal laser microscope (Leica TCS SP8, Leica Microsystems, Wetzlar, Germany). Glomerular CD31-positive staining was quantified in 15 glomeruli per section, expressing the positive glomerular areas as a percentage of the total area (ImageJ software, 2.3.0/1.53q, NIH, Bethseda, MD, USA).

### 4.7. Transmission Electron Microscopy Analysis

Kidney tissue fragments were fixed overnight in 2.5% glutaraldehyde (catalog 340855 Sigma Aldrich, Darmstadt, Germany) in a 0.1 mol/L cacodylate buffer (pH 7.4) (catalog 11652, Electron Microscopy Sciences, Hatfield, PA, USA) and washed repeatedly in the same buffer (*n* = 3 mice/each group). After postfixation in 1% OsO_4_, specimens were dehydrated through ascending grades of alcohol and embedded in epon resin. Ultrathin sections were stained with UranyLess (catalog 22409, UAR, Electron Microscopy Sciences) and lead citrate (catalog 22410, Electron Microscopy Sciences) and examined using a transmission electron microscope (Morgagni 268D; Philips, Brno, Czech Republic).

### 4.8. Citrate Synthase Activity

Kidney samples were washed twice in 0.9% (*w*/*v*) sodium chloride solution and homogenized in a CelLytic MT buffer (C3228; Sigma-Aldrich, St. Louis, MO, USA) supplemented with protease inhibitor cocktail (P8340; Sigma-Aldrich), and tissue disruption was completed by using a blunt-ended needle and a syringe. The lysates were centrifuged 16,000× *g* for 10 min at 4 °C, and supernatants were collected. The total protein concentration was determined using a Pierce™ BCA Protein Assay Kit (23227; ThermoFisher, Waltham, MA, USA). Ten micrograms of proteins was analysed by a citrate synthase activity assay (CS072; Sigma-Aldrich) according to the manufacturer’s protocol. The activity of citrate synthase was assessed by using the multimode microplate reader TECAN Infinite M200^®^ PRO (Tecan Group Ltd., Mannedorf, Switzerland) at 412 nm under a controlled temperature with a kinetic program.

### 4.9. Statistical Analysis

The results were expressed as mean ± SEM. Data analyses were performed using Graph Pad Prism software (Graph Pad, San Diego, CA, USA). Comparisons were performed using one-way ANOVA with Tukey’s multiple comparisons post hoc test or Student’s *t* test where appropriate, and the statistical significance was defined as a *p* value of <0.05.

## Figures and Tables

**Table 1:** Laboratory and systemic parameters in WT and *Sirt3*^−/−^ mice fed with a standard or high-fat-diet.

Groups	Plasma Cholesterol (mg/dL)	Plasma Triglycerides (mg/dL)	SBP (mmHg)	Heart Rate (beats/min)	BUN (mg/dL)
WT + STD	102.3 ± 1.8	123.9 ± 9.2	103.4 ± 2.3	561.1 ± 16.3	22.4 ± 1.1
	(*n* = 10)	(*n* = 10)	(*n* = 10)	(*n* = 10)	(*n* = 10)
*Sirt3*^−/−^ + STD	106.4 ± 3.1	129.8 ± 3.8	111.9 ± 2.2	564.3 ± 22.2	20.3 ± 1.1
	(*n* = 12)	(*n* = 12)	(*n* = 12)	(*n* = 12)	(*n* = 12)
WT + HFD	128.7 ± 5.1 *	154.5 ± 5.4 *	113.4 ± 4.7	590.1 ± 11.8	19.8 ± 1.1
	(*n* = 10)	(*n* = 10)	(*n* = 10)	(*n* = 10)	(*n* = 10)
*Sirt3*^−/−^ + HFD	139.3 ± 6.2 **	144.4 ± 4.2	109.6 ± 3.4	651.3 ± 15.6 *^#^	20.7 ± 0.9
	(*n* = 14)	(*n* = 14)	(*n* = 14)	(*n* = 14)	(*n* = 14)

Data are mean ± SEM. * *p* < 0.01, ** *p* < 0.0001 vs. corresponding groups with STD (one way ANOVA); ^#^ *p <* 0.01 vs. WT + HFD (unpaired Student’s *t*-test). Abbreviations: STD, standard diet; HFD, high-fat-diet; SBP, systolic blood pressure; BUN, blood urea nitrogen.

## Data Availability

All data generated in the study are presented in the manuscript.
